# *LRRK2* rare-variant per-domain genetic burden in Parkinson’s Disease: association confined to the kinase domain

**DOI:** 10.1038/s41531-025-00934-z

**Published:** 2025-04-29

**Authors:** Sitki Cem Parlar, Konstantin Senkevich, Eric Yu, Jennifer A. Ruskey, Jamil Ahmad, Farnaz Asayesh, Dan Spiegelman, Cheryl Waters, Oury Monchi, Yves Dauvilliers, Nicolas Dupré, Lior Greenbaum, Sharon Hassin-Baer, Irina Miliukhina, Alla Timofeeva, Anton Emelyanov, Sofya Pchelina, Roy N. Alcalay, Edward A. Fon, Jean-François Trempe, Ziv Gan-Or

**Affiliations:** 1 Department of Human Genetics, McGill University, Montréal, QC Canada; 2 The Neuro (Montreal Neurological Institute-Hospital), McGill University, Montréal, QC Canada; 3 Department of Neurology and Neurosurgery, McGill University, Montréal, QC Canada; 4 Department of Specialized Medicine, Division of Medical Genetics, McGill University Health Centre, Montreal, QC Canada; 5 Department of Neurology, College of Physicians and Surgeons, Columbia University Medical Center, New York, NY USA; 6 Department of Clinical Neurosciences and Department of Radiology, University of Calgary, Calgary, AB Canada; 7 Hotchkiss Brain Institute, Cumming School of Medicine, Calgary, AB Canada; 8 National Reference Center for Narcolepsy, Sleep Unit, Department of Neurology, Gui-de-Chauliac Hospital, CHU Montpellier, University of Montpellier, Montpellier, France; 9 Division of Neurosciences, CHU de Québec, Université Laval, Quebec City, QC Canada; 10 Department of Medicine, Faculty of Medicine, Université Laval, Québec, QC Canada; 11 The Danek Gertner Institute of Human Genetics, Sheba Medical Center, Ramat Gan, Israel; 12 The Joseph Sagol Neuroscience Center, Sheba Medical Center, Ramat Gan, Israel; 13 Faculty of Medicine, Tel Aviv University, Tel Aviv-Yafo, Israel; 14 The Movement Disorders Institute, Department of Neurology, Sheba Medical Center, Tel Aviv, Israel; 15 Institute of the Human Brain of RAS, Saint Petersburg, Russia; 16 First Pavlov State Medical University of St. Petersburg, Saint Petersburg, Russia; 17 Division of Movement Disorders, Tel Aviv Sourasky Medical Center, Tel Aviv-Yafo, Israel; 18 Early Drug Discovery Unit (EDDU), Montreal Neurological Institute-Hospital (The Neuro), Montreal, QC Canada; 19 Department of Pharmacology & Therapeutics and Centre de Recherche en Biologie Structurale, McGill University, Montréal, QC Canada

**Keywords:** Genetics, Parkinson's disease

## Abstract

*LRRK2* variants are key genetic risk factors for Parkinson’s Disease (PD). We conducted a per-domain rare coding variant burden analysis, including 8,888 PD cases and 69,412 controls. In meta-analysis, the Kinase domain was strongly associated with PD (Exonic: *P*^*FDR*^ = 1.61 × 10^−22^, Non-synonymous: *P*^*FDR*^ = 1.54 × 10^−23^, CADD > 20: *P*^*FDR*^ = 3.09 × 10^−24^). Excluding the p.G2019S variant nullified this effect. Nominal associations were found in the ANK and Roc-COR domains, with potentially protective variants, p.R793M and p.Q1353K.

## Introduction

Leucine-rich repeat kinase 2 (*LRRK2*) protein is part of the endolysosomal-autophagy pathway, where numerous proteins are implicated in PD pathogenesis^1^. Variants in the *LRRK2* gene have been associated with both familial and sporadic forms of PD, yet the specific pathogenic mechanism underlying the association between *LRRK2* and PD is unclear^2^. The most common pathogenic variant, p.G2019S, is associated with increased risk of developing PD of up to 10-fold in certain populations^3,4^, with evidence for also affecting the prevalence of dystonia in PD^5^.

**Fig. 1:**
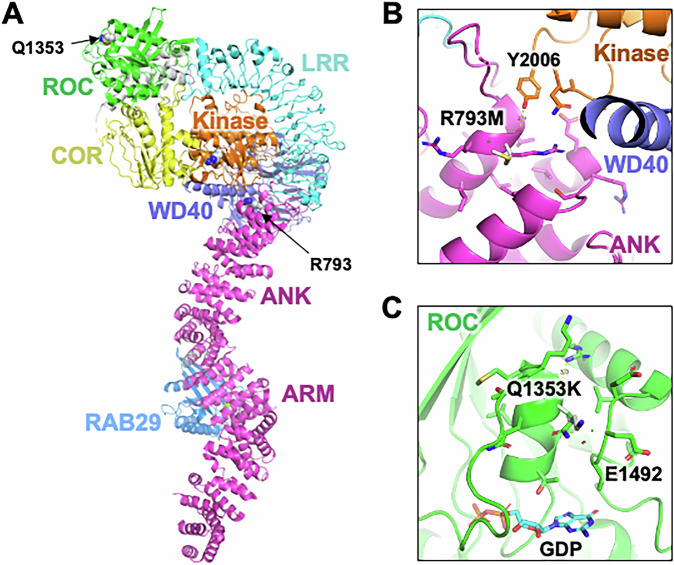
Structural analysis of PD variants in LRRK2. **A** Cryoelectron microscopy structure of LRRK2, PDB code 8FO2^15^. The location of Parkinson-linked missense mutation sites p.R793M and p.Q1353K are indicated. **B**, **C** Details of the impact of mutations listed above, as described in the text.

The structure of the *LRRK2* protein consists of seven domains, including Armadillo repeat (ARM), Ankyrin repeat (ANK), Leucine-rich repeat (LRR), Kinase, Ras-of-complex (Roc) GTPase in tandem with C-terminal of Roc (COR), and WD40 (Fig. 1). The domains can be grouped by functions: (1) ARM, ANK, LRR, and WD40 serve as scaffolding domains, (2) Kinase domain contains the catalytic site for kinase activity, and (3) the Roc-COR bidomain contains the catalytic site for the GTPase^6^.

The *LRRK2* Kinase and Roc-COR regions are of particular interest as they contain catalytic functions of the protein. Most of the known pathogenic variants are in these two sites^7^, most of which have been shown to alter kinase enzymatic activity^8^. For instance, the pathogenic p.G2019S variant is located in the Kinase domain, and leads to increased kinase activity^9^, suggesting that *LRRK2* Kinase inhibition could be a therapeutic strategy for PD^8^. Another pathogenic variant, p.R1441H, is located in the Roc-COR bidomain region and is associated with altered GTPase function^10^. While evidence for pathogenicity outside of these two catalytic sites is limited, the PD risk variant p.G2835R, located in the WD40 domain, has been shown to increase disease risk by two-fold in East Asians^11^, with in vitro evidence for increase of *LRRK2* Kinase activity^12^.

The association between *LRRK2* rare variants and PD risk burden has been established^13^. However, the association of specific *LRRK2* domains burden in PD risk is not known. Domain-level analysis could aid in the identification of novel variants and provide functional insight. In this paper, across six cohorts, we meta-analyzed the PD burden of *LRRK2* rare variants using the optimized sequence kernel association test (SKAT-O) per-domain in 8,888 PD cases and 69,412 controls (detailed in Supplementary Data 1). We then conducted structural analyzes of variants of potential significance to understand how certain variants could be altering the structure, function, and interactions of the protein.

The average coverage across the four cohorts sequenced locally at McGill exceeded 652X, with over 99% of nucleotides achieving coverage greater than 30× (Supplementary Data 2). Across the four functional categories (exonic, non-synonymous, Combined Annotation Dependent Depletion (CADD) score ≥ 20, and loss-of-function (LOF)) and after quality control steps were applied, in our local cohorts, we analyzed 105 unique variants from the McGill University cohort, 65 unique variants from the Columbia University cohort, 47 unique variants from the Sheba Medical Center cohort, and 113 unique variants from the Pavlov First State Medical University and Institute of Human Brain cohort. In addition to this list of variants, from the external cohorts, 454 unique variants from United Kingdom Biobank (UKBB) and 96 unique variants from Accelerating Medicines Partnership–Parkinson Disease (AMP-PD) were also analyzed. The complete list of these variants is provided in Supplementary Data 3 and 4 for the local and external cohorts, respectively.

The per-domain burden analysis showed a post-False Discovery Rate (FDR) significance in the *LRRK2* Kinase domain regions in the SKAT-O results across five of the six cohorts (Supplementary Data 5) and the MetaSKAT results of the meta-analysis (See Table 1 and, for more detail, Supplementary Data 6). We then performed SKAT-O and MetaSKAT analyzes after excluding the p.G2019S variant, resulting in a complete elimination of association in the *LRRK2* Kinase domain (Table 2). In this domain, the p.G2019S variant was the driver of association, and in its absence, the aggregate of the remaining variants in the region could not drive a significant association for PD risk.

**Table 1:** Meta-analysis results of per-domain rare variant burden in LRRK2

Domain	Exonic variants, *P*^*FDR*^	Non-synonymous (missense only), *P*^*FDR*^	CADD ≥ 20, *P*^*FDR*^	LOF, *P*^*FDR*^
ARM	0.55	0.85	0.80	0.80
ANK	0.26	0.15	0.57	0.85
LRR	0.92	0.90	0.80	0.85
Roc-COR	0.15	0.15	0.26	0.80
Kinase	1.61 × 10^-22^	1.54 × 10^-23^	3.09 × 10^-24^	0.31
WD40	0.80	0.80	0.24	1

*P* *P* value, *FDR* false discovery rate, *CADD* Combined Annotation Dependent Depletion score, *LOF* loss-of-function.

**Table 2:** SKAT-O and MetaSKAT analysis of LRRK2 Kinase rare variants with and without p.G2019S

	Kinase with p.G2019S				Kinase without p.G2019S			
Cohort	Exonic variants, *P*^*FDR*^	Non-synonymous (missense only), *P*^*FDR*^	CADD ≥ 20, *P*^*FDR*^	LOF, *P*^*FDR*^	Exonic variants, *P*^*FDR*^	Non-synonymous (missense only), *P*^*FDR*^	CADD ≥ 20, *P*^*FDR*^	LOF, *P*^*FDR*^
Columbia University cohort	1.74 × 10^−7^	2.89 × 10^−8^	2.89 × 10^−8^	-	0.96	0.96	-	-
Sheba Medical Centre cohort	4.55 × 10^−4^	4.55 × 10^−4^	4.55 × 10^−4^	-	0.63	0.99	-	-
McGill University cohort	1.21 × 10^−5^	1.93 × 10^−5^	3.12 × 10^−5^	-	0.39	0.42	0.59	-
Pavlov and Human Brain cohort	0.67	0.67	0.67	-	0.90	0.73	0.73	-
UKBB	1.29 × 10^−8^	1.29 × 10^−8^	2.46 ×10^−8^	0.91	0.91	0.91	0.91	0.91
AMP-PD	8.68 × 10^−6^	5.11 × 10^−6^	5.11 ×10^−6^	0.30	0.67	0.82	0.42	0.42
Meta-analysis	1.61 × 10^−22^	1.54 × 10^−23^	3.09 × 10^−24^	0.31	1	1	0.42	0.42

*P* *P* value, *FDR* false discovery rate, *CADD* combined annotation dependent depletion score, *LOF* loss-of-function, *UKBB* United Kingdom Biobank, *AMP-PD* Accelerating Medicines Partnership–Parkinson Disease.

In other domains, the associations did not survive the correction for multiple comparisons. However, nominal significance was observed in: (1) the ARM domain in the Pavlov cohort (Exonic: *P* = 0.04), (2) the ANK domain in the McGill cohort (Non-synonymous: *P* = 0.03) and the meta-analysis (Non-synonymous: *P* = 0.03), (3) the Roc-COR domain region in UKBB (Exonic: *P* = 0.01; Non-synonymous: *P* = 0.01; CADD ≥ 20: *P* = 0.04) and the meta-analysis (Exonic: *P* = 0.04; Non-synonymous: *P* = 0.04).

We nominated two variants p.R793M and p.Q1353K to be potentially protective, based on their frequencies in patients and controls. While these variants showed intriguing patterns, we did not observe statistically significant odds ratios (OR) values to affirm our observations. The nominated variants with their sample and frequency data across all cohorts and The Genome Aggregation Database (gnomAD) non-Finnish European are detailed in Table 3.

**Table 3:** Reported samples and frequencies of nominated potentially protective *LRRK2* rare variants

Cohorts	McGill University		Columbia University		Sheba		Pavlov and Human Brain		UKBB		AMP-PD		gnomAD v4.1 NFE
Variant	Cases N, (MAF)	Controls (MAF)	Cases N, (MAF)	Controls (MAF)	Cases N, (MAF)	Controls (MAF)	Cases N, (MAF)	Controls (MAF)	Cases N, (MAF)	Controls (MAF)	Cases N, (MAF)	Controls (MAF)	MAF
p.R793M	0	9 (0.001839)	1 (0.0003984)	0	-	-	0	1 (0.001553)	-	-	0	2 (0.0003233)	0.0008
p.Q1353K	0	8 (0.001635)	-	-	-	-	-	-	-	-	1 (0.0002547)	2 (0.0003233)	0.0001

*N* number of samples, *MAF* minor allele frequency, *UKBB* United Kingdom Biobank, *AMP-PD* Accelerating Medicines Partnership–Parkinson Disease, *gnomAD* the genome aggregation database, *NFE* Non-Finnish European

In our variant-level OR calculations, we did not observe any p-value significance except for p.G2019S (See Supplementary Data 3 and 4). Certain variants in UKBB were also marked as statistically significant in continuity corrected OR calculations; however, this significance is attributed to the disproportionate 1:20 case-to-control ratio in UKBB, which biases the corrected results. Consequently, UKBB variants were disregarded in the nomination process.

The ANK domain showed nominal significance in the burden analysis in the McGill cohort and the meta-analysis. Located in this region, the rare variant p.R793M was nominated for its presence in 9 controls and none in patients in the McGill cohort, suggesting potential protective association. The exclusion of p.R793M from the burden analysis resulted in the loss of the nominal significance in the ANK domain (See Supplementary Data 5 and 6), showing that the p.R793M was driving the nominal regional association of its domain in McGill cohort and the meta-analysis.

Secondly, *LRRK2* Roc-COR bidomain showed nominal significance in the meta-analysis, which potentially implicates the p.Q1353K variant, as it was reported only in 8 controls in the McGill University. It was also reported in AMP-PD in 1 case and 2 controls. This variant was noted previously before in the McGill University cohort^14^ with no significant variant-level post-correction associations. At domain level, we were also unable to observe post-correction significance. In addition, the exclusion of p.Q1353K from the burden analysis did not nullify any nominal association, which limits the evidence for its protective role in PD to being solely observational (See Supplementary Data 5 and 6).

The influence of these two variants was also studied structurally to better understand how they may affect the function of the LRRK2 protein and its domains.

*LRRK2* can be recruited to biological membranes of organelles, where it can be activated by RAB29 binding to the ARM domain^15^. RAB29 binding is accompanied by formation of tetramer, where two *LRRK2* molecules occupy the center and adopt an active Kinase conformation, whereas the two peripheral protomers are bound to RAB29 and adopt an inactive Kinase conformation. These conformations are regulated by allosteric interactions between the different domains (inactive full-length protomer shown in Fig. 1A). Furthermore, oligomerization and allosteric changes also regulate interactions with microtubules^6,16^. To gain insight into the functional effects of missense variants in *LRRK2*, we analyzed them in the structure of human *LRRK2* in complex with the small GTPase RAB29.

The first observation is that the identified protective variants are not at oligomerization interfaces. The p.R793M variant, which is potentially protective, is in the ANK, near the interface with the Kinase and WD40 domains (Fig. 1B). Arginine is a positively charged residue, and thus the change to Methionine would reduce polar interactions with the Kinase domain, and thus could modulate the coupling between RAB29 or microtubule binding and Kinase activation. The p.Q1353K variant, again potentially protective, is in the ROC GTPase domain and is located near the nucleotide binding site (Fig. 1C). The variant would introduce a positive charge, which could create a new interaction with p.E1492 on the opposite side, thus affecting the GTPase activity. These variants may protect from PD by increasing the threshold for activation through allostery, thus reducing the overall activity of the protein (without abolishing it). The p.R793M variant was indeed reported to decrease MLi-2-induced microtubule association, while not significantly affecting the intrinsic Kinase activity^12^.

In our per-domain analysis, we showed, as expected, a significant association between the Kinase domain of *LRRK2* and PD. We did not find any secondary signal after excluding the p.G2019S variant in the Kinase domain. However, it is important to note that there are other, much rarer variants in this domain that are known to cause PD and were too rare in our tested populations to be identified, such as p.I2020T^17^. While other associations in other domains did not survive the FDR corrections, nominal associations were observed (See Supplementary Data 5 and 6), with the ANK and Roc-COR domain region rare variants showing domain-level nominal significance in the meta-analysis.

In closer inspection, we nominated two potentially protective variants: p.R793M and p.Q1353K, based on evidence from the burden tests, population frequencies, and number of samples carrying them in cases vs. controls. We further examined the potential structural effects of these variants in simulations, yet we cannot conclusively determine whether they have a protective role in PD. The well-established major risk variant *LRRK2* p.G2019S has been shown to increase Kinase activity. This is supportive of a gain-of-function mechanism for the association between *LRRK2* and PD risk. It has been previously suggested that inhibiting Kinase activity could help alleviate PD pathology^8^, and safety of an *LRRK2* kinase-inhibiting drug has been tested in a human clinical trial^18^.

*LRRK2* interacts with a variety of Rab-based GTPase proteins^19^, which are increasingly implicated in autophagy-related disorders. Recently, the RAB32 protein has been identified as a novel PD susceptibility gene^20^. *LRRK2* interacts with RAB32 through its ANK domain^21^. Rare variants in *LRRK2* ARM and ANK domains could influence the affinities of binding with the Rab-family of proteins. However, the evidence as to how this could influence PD risk is unclear. Amongst the two variants we have nominated, cell assays studying the effect of p.R793M could be insightful in showing whether this variant protects against PD pathobiology.

Our study was limited by various factors. Different quality control standards were applied for targeted sequencing, whole-exome sequencing, and whole-genome sequencing data, using varying thresholds for depth of coverage and quality assessment as per platform recommendations. This variability could potentially lead to discrepancies in the enrichment of variants between different cohorts. Particularly, the UKBB dataset was subject to lower GQ and depth of coverage thresholds. Another constraint was the absence of a conventional power calculation in our statistical analysis. Unlike methods that rely on clear effect size estimates, SKAT-O primarily evaluates genetic effects in terms of variance components, which do not readily translate into parameters suitable for standard power estimation. As a result, we could not accurately gauge the statistical power. The lack of diversity in the populations that were included presented another limitation. All of our six cohorts mainly consisted of European populations, which may have led to us missing important *LRRK2* variants. These included the risk-inducing p.R1628P and p.G2835R variants, located in the Roc-COR and WD40 domain regions, respectively, and predominantly found in East Asian populations^4,11^. Specifically, the p.G2835R was absent across all our cohorts. For future studies, we recognize the importance of incorporating more diverse ethnic populations to allow for a more comprehensive set of variants and to improve our ability to detect regional risk associations, such as at gene-level or domain-level, as is the case in this study. In addition, variants that are known to be risk factors for PD, such as p.N2081D, were excluded since their MAF in Europeans is higher than 0.01.

Overall, our study tests the potential roles of each domain of the *LRRK2* protein, yet insight on the potential roles of the different *LRRK2* domains in PD remains limited, with the nomination of two potentially protective variants that require further work to substantiate any functional implications.

## Methods

### Study population

In this study, we include six large cohorts with a total of 8888 PD patients and 69,412 controls (further detailed in Supplementary Data 1), post-quality control. Clinical diagnosis was performed by movement disorder specialists following the UK brain bank criteria^22^ or the movement disorders clinical diagnostic criteria^23^. Four distinct cohorts were collected and sequenced at McGill University: the McGill cohort (comprising participants from Quebec, Canada, and Montpellier, France), the Columbia University cohort (New York, NY), the Sheba Medical Center cohort (Israel), and the Pavlov First State Medical University and Institute of Human Brain cohort (Saint-Petersburg, Russia). The McGill cohort included participants from Quebec, Canada, recruited partly through the Quebec Parkinson Network (QPN)^24^, and from France. The Columbia cohort, collected in New York, represented a mixed ancestry group, including individuals of European descent, Ashkenazi Jewish (AJ) background, and a smaller proportion of Hispanic and Black participants, as described previously^25^. The Sheba cohort, recruited in Israel, consisted of participants with AJ ancestry, as reported previously^26^ as well as non-Ashkenazi Jewish ancestry. The Pavlov and Human Brain cohort, recruited in Russia, primarily included patients of European descent. Additionally, two external patient cohorts from UKBB and AMP-PD, which mainly consist of European samples, were accessed and analyzed (See Supplementary Data 1).

### Data sequencing

We sequenced the four cohorts (McGill, Columbia, Sheba, and Pavlov) at McGill University via a next-generation targeted panel sequencing approach whereby the coding and non-coding regions of *LRRK2* were targeted using molecular inversion probes (MIPs) as previously described^27^. The probes used to target *LRRK2* can be found in Supplementary Data 7 along with the full protocol at https://github.com/gan-orlab/MIP_protocol. The Illumina NovaSeq 6000 SP PE100 platform at the Genome Quebec Innovation Centre was used for the sequencing of the library. The sequenced data were aligned to the hg19 reference genome using the Burrows-Wheeler Aligner^28^.

As for the external cohorts, the UKBB whole-exome sequencing data was sequenced using the Illumina NovaSeq 6000 platform S2 and S4, and the AMP-PD whole-genome sequencing data was sequenced using the Illumina HiSeq X Ten platform, both being aligned to the hg38 reference genome^29,30^. We realigned our local cohorts from hg19 to hg38 to have consistency across all cohorts using CrossMap^31^. Finally, we extracted the part of the genome encoding for the *LRRK2* gene at chr12:40224997- 40369285 for quality control and analysis.

### Data quality control

We applied quality control steps at both sample and variant levels. In all cohorts, any first- and second-degree relatives were removed, keeping only unrelated samples. In UKBB, we applied an additional step to remove non-European samples based on UKBB data field 21,000. This step was also applied for AMP-PD where non-European samples were identified and excluded using admixture analysis, where we employed reference populations to detect genetic ancestry and ensure the exclusion of samples of non-European ancestry. Later, we used the genome analysis toolkit (GATK) for filtering based on minimum genotype quality (GQ) score and depth of coverage^32^. For the four cohorts sequenced at McGill University, GATK was used to apply thresholds of GQ > 25 and depth of coverage > 30x and to remove variants with genotyping rate of less than 90%. The sample depth of coverage data was averaged for *LRRK2* for each of the four cohorts sequenced at McGill University. For UKBB whole-exome sequencing data, using GATK, thresholds were set for GQ > 20, depth of coverage > 10x, and genotyping rate > 95%, leaving only high-quality samples and variants. Applying the same stringent quality control parameters used for the McGill cohorts was not feasible for the UKBB due to differences in sequencing methods, and as it would lead to substantial loss of data for the latter. AMP-PD whole-genome sequencing data were pre-filtered by AMP-PD with steps detailed on their website (https://amp-pd.org/whole-genome-data) and in a previous study^30^. Across all cohorts, a minor allele frequency (MAF) threshold of 0.01 was applied to include only rare variants using plink v1.9^33^, and multi-allelic variants were excluded using bcftools^34^. Exceptionally, we kept p.G2019S pathogenic variant in all cohorts (it has MAF of >0.01 in AJ) to understand the effect of its presence vs. absence on the *LRRK2* Kinase burden for PD.

### Domain demarcation

For domain-specific analysis, we demarcated the domains of *LRRK2* based on the domain annotation resources available online at Ensembl^35^ (https://www.ensembl.org): PANTHER, Superfamily, Gene3D, Smart, Pfam, and Prosite^36–41^. The widest range was taken based on the aggregation of domain annotation data, and the genomic intervals were established for the following six domain regions: ARM, ANK, LRR, Roc-COR, Kinase, and WD40 (See Supplementary Data 8).

### Variant annotation

As the study was particularly interested in studying *LRRK2* domains, variants that were only located within these domain regions were included. We annotated variants using ANNOVAR^42^ with functional information, including the deleteriousness scores based on the Combined Annotation Dependent Depletion (CADD) v1.6^43^. For further population-level insights, The Genome Aggregation Database (gnomAD) v4.1 was also used in the annotation process to match variants with MAF data across various populations^44^.

### Burden analysis

To understand how functionally distinct variant groups could drive association differentially, we stratified variants into four categories: (1) Exonic: all exonic variants, (2) non-synonymous: all non-synonymous protein-change variants, (3) CADD ≥ 20: top 1% of potentially deleterious variants, and (4) Loss-of-function (LOF): all stopgain, frameshift and splice-site variants on splicing acceptor and donor sites (+1/−1 and +2/−2 positions). The four categories of *LRRK2* variants were analyzed per-domain for their burden for PD via SKAT-O^45^. Further, MetaSKAT^46^ was used to meta-analyze all six cohorts. We included sex and age as covariates to account for potential confounding effects associated with these variables. The minimize the likelihood of false-positive findings, false discovery rate (FDR) corrections were applied to the results^47^.

### Variant nomination

Restricting our investigation to domains that achieved at least nominal significance in either cohort-specific or meta-analytic burden tests, we conducted an observational assessment of non-synonymous variant frequencies among cases and controls. Variants were shortlisted and additionally evaluated with an Odds Ratio (ORs) analysis, both with and without continuity corrections. The continuity correction was applied by adding 0.5 to both cases and controls. Based on our observations, variants were nominated for subsequent structural analysis.

### In silico structural analysis

The atomic coordinates of human *LRRK2* were downloaded from the Protein Data Bank (ID 8FO2). The figure was generated using PyMol v.3.0.4.

## Supplementary information

Supplementary Information

## Data Availability

The datasets used in this study include those from the AMP-PD Knowledge Platform (https://www.amp-pd.org) and the UKBB, both of which require institutional approvals for access. Additionally, data from local cohorts sequenced at McGill University are currently restricted to internal use and collaborations as the informed consent forms do not allow for public sharing of genetic data. All data directly relevant to this study’s analysis, including the list of analyzed variants and demographics of the study population, are provided in the supplementary files of this article.

## Code availability

The bioinformatics pipelines and scripts used in this study can be found in the study’s GitHub repository at: https://github.com/gan-orlab/LRRK2_per_domain.

## References

[1] Lynch-Day, M. A., Mao, K., Wang, K., Zhao, M. & Klionsky, D. J. The role of autophagy in Parkinson’s disease. *Cold Spring Harb. Perspect. Med.* **2**, a009357 (2012). 22474616 10.1101/cshperspect.a009357 PMC3312403

[2] Zimprich, A. et al. Mutations in LRRK2 cause autosomal-dominant parkinsonism with pleomorphic pathology. *Neuron* **44**, 601–607 (2004). 15541309 10.1016/j.neuron.2004.11.005

[3] Iwaki, H. et al. Penetrance of Parkinson’s disease in LRRK2 p.G2019S carriers is modified by a polygenic risk score. *Mov. Disord.* **35**, 774–780 (2020). 31958187 10.1002/mds.27974 PMC8975556

[4] Simpson, C. et al. Prevalence of ten LRRK2 variants in Parkinson’s disease: a comprehensive review. *Parkinsonism Relat. Disord.* **98**, 103–113 (2022). 35654702 10.1016/j.parkreldis.2022.05.012

[5] Bouhouche, A. et al. LRRK2 G2019S mutation: prevalence and clinical features in moroccans with Parkinson’s disease. *Parkinsons Dis.* **2017**, 2412486 (2017). 28465860 10.1155/2017/2412486 PMC5390546

[6] Deniston, C. K. et al. Structure of LRRK2 in Parkinson’s disease and model for microtubule interaction. *Nature* **588**, 344–349 (2020). 32814344 10.1038/s41586-020-2673-2 PMC7726071

[7] Sosero, Y. L. & Gan-Or, Z. LRRK2 and Parkinson’s disease: from genetics to targeted therapy. *Ann. Clin. Transl. Neurol.* **10**, 850–864 (2023). 37021623 10.1002/acn3.51776 PMC10270275

[8] Taymans, J. M. & Greggio, E. LRRK2 kinase inhibition as a therapeutic strategy for Parkinson’s Disease, where do we stand?. *Curr. Neuropharmacol.* **14**, 214–225 (2016). 26517051 10.2174/1570159X13666151030102847 PMC4857626

[9] Rui, Q., Ni, H., Li, D., Gao, R. & Chen, G. The role of LRRK2 in neurodegeneration of Parkinson's disease. *Curr. Neuropharmacol.* **16**, 1348–1357 (2018). 29473513 10.2174/1570159X16666180222165418 PMC6251048

[10] Liao, J. et al. Parkinson disease-associated mutation R1441H in LRRK2 prolongs the “active state” of its GTPase domain. *Proc. Natl. Acad. Sci. USA* **111**, 4055–4060 (2014). 24591621 10.1073/pnas.1323285111 PMC3964117

[11] Shu, L., Zhang, Y., Sun, Q., Pan, H. & Tang, B. A comprehensive analysis of population differences in LRRK2 variant distribution in Parkinson’s disease. *Front. Aging Neurosci.* **11**, 13 (2019). 30760999 10.3389/fnagi.2019.00013 PMC6363667

[12] Kalogeropulou, A. F. et al. Impact of 100 LRRK2 variants linked to Parkinson’s disease on kinase activity and microtubule binding. *Biochem. J.* **479**, 1759–1783 (2022). 35950872 10.1042/BCJ20220161 PMC9472821

[13] Makarious, M. B. et al. Large-scale rare variant burden testing in Parkinson’s disease. *Brain* **146**, 4622–4632 (2023). 37348876 10.1093/brain/awad214 PMC10629770

[14] Rudakou, U. et al. Targeted sequencing of Parkinson’s disease loci genes highlights SYT11, FGF20 and other associations. *Brain* **144**, 462–472 (2021). 33349842 10.1093/brain/awaa401 PMC7940168

[15] Zhu, H. et al. Rab29-dependent asymmetrical activation of leucine-rich repeat kinase 2. *Science* **382**, 1404–1411 (2023). 38127736 10.1126/science.adi9926 PMC10786121

[16] Watanabe, R. et al. The in situ structure of Parkinson’s disease-linked LRRK2. *Cell* **182**, 1508–1518.e1516 (2020). 32783917 10.1016/j.cell.2020.08.004 PMC7869717

[17] Pitz, V. et al. Analysis of rare Parkinson’s disease variants in millions of people. *NPJ Parkinsons Dis.* **10**, 11 (2024). 38191580 10.1038/s41531-023-00608-8 PMC10774311

[18] Jennings, D. et al. LRRK2 inhibition by BIIB122 in healthy participants and patients with Parkinson’s disease. *Mov. Disord.* **38**, 386–398 (2023). 36807624 10.1002/mds.29297

[19] Kuwahara, T. & Iwatsubo, T. The emerging functions of LRRK2 and Rab GTPases in the endolysosomal system. *Front. Neurosci.* **14**, 227 (2020). 32256311 10.3389/fnins.2020.00227 PMC7095371

[20] Hop, P. J. et al. Systematic rare variant analyses identify RAB32 as a susceptibility gene for familial Parkinson’s disease. *Nat. Genet* **56**, 1371–1376 (2024). 38858457 10.1038/s41588-024-01787-7 PMC11250361

[21] McGrath, E., Waschbusch, D., Baker, B. M. & Khan, A. R. LRRK2 binds to the Rab32 subfamily in a GTP-dependent manner via its armadillo domain. *Small GTPases* **12**, 133–146 (2021). 31552791 10.1080/21541248.2019.1666623 PMC7849779

[22] Hughes, A. J., Ben-Shlomo, Y., Daniel, S. E. & Lees, A. J. What features improve the accuracy of clinical diagnosis in Parkinson’s disease: a clinicopathologic study. *Neurology* **42**, 1142–1146 (1992). 1603339 10.1212/wnl.42.6.1142

[23] Postuma, R. B. et al. MDS clinical diagnostic criteria for Parkinson’s disease. *Mov. Disord.* **30**, 1591–1601 (2015). 26474316 10.1002/mds.26424

[24] Gan-Or, Z. et al. The Quebec Parkinson Network: A Researcher-Patient Matching Platform and Multimodal Biorepository. *J. Parkinsons Dis.* **10**, 301–313 (2020). 31868683 10.3233/JPD-191775 PMC7029361

[25] Alcalay, R. N. et al. SCARB2 variants and glucocerebrosidase activity in Parkinson’s disease. *NPJ Parkinsons Dis.* **2**, 16004 (2016). 27110593 10.1038/npjparkd.2016.4 PMC4838276

[26] Ruskey, J. A. et al. Increased yield of full GBA sequencing in Ashkenazi Jews with Parkinson’s disease. *Eur. J. Med. Genet.* **62**, 65–69 (2019). 29842932 10.1016/j.ejmg.2018.05.005 PMC6261782

[27] Rudakou, U. et al. Analysis of common and rare VPS13C variants in late-onset Parkinson disease. *Neurol. Genet.* **6**, 385 (2020). 32042909 10.1212/NXG.0000000000000385 PMC6984134

[28] Li, H. & Durbin, R. Fast and accurate short read alignment with Burrows-Wheeler transform. *Bioinformatics* **25**, 1754–1760 (2009). 19451168 10.1093/bioinformatics/btp324 PMC2705234

[29] Backman, J. D. et al. Exome sequencing and analysis of 454,787 UK Biobank participants. *Nature* **599**, 628–634 (2021). 34662886 10.1038/s41586-021-04103-z PMC8596853

[30] Iwaki, H. et al. Accelerating Medicines Partnership: Parkinson’s disease. Genetic resource. *Mov. Disord.* **36**, 1795–1804 (2021). 33960523 10.1002/mds.28549 PMC8453903

[31] Zhao, H. et al. CrossMap: a versatile tool for coordinate conversion between genome assemblies. *Bioinformatics* **30**, 1006–1007 (2014). 24351709 10.1093/bioinformatics/btt730 PMC3967108

[32] Van der Auwera, G. A. & O’Connor, B. D. *Genomics in the cloud: using Docker, GATK, and WDL in Terra* (O’Reilly Media, 2020).

[33] Chang, C. C. et al. Second-generation PLINK: rising to the challenge of larger and richer datasets. *Gigascience* **4**, 7 (2015). 25722852 10.1186/s13742-015-0047-8 PMC4342193

[34] Danecek, P. et al. Twelve years of SAMtools and BCFtools. *Gigascience* **10**, 10.1093/gigascience/giab008 (2021). 10.1093/gigascience/giab008 PMC7931819 33590861

[35] Harrison, P. W. et al. Ensembl 2024. *Nucleic Acids Res.* **52**, D891–D899 (2024). 37953337 10.1093/nar/gkad1049 PMC10767893

[36] El-Gebali, S. et al. The Pfam protein families database in 2019. *Nucleic Acids Res.* **47**, D427–D432 (2019). 30357350 10.1093/nar/gky995 PMC6324024

[37] Lees, J. et al. Gene3D: a domain-based resource for comparative genomics, functional annotation and protein network analysis. *Nucleic Acids Res.* **40**, D465–D471 (2012). 22139938 10.1093/nar/gkr1181 PMC3245158

[38] Letunic, I. & Bork, P. 20 years of the SMART protein domain annotation resource. *Nucleic Acids Res.* **46**, D493–D496 (2018). 29040681 10.1093/nar/gkx922 PMC5753352

[39] Pandurangan, A. P., Stahlhacke, J., Oates, M. E., Smithers, B. & Gough, J. The SUPERFAMILY 2.0 database: a significant proteome update and a new webserver. *Nucleic Acids Res.* **47**, D490–D494 (2019). 30445555 10.1093/nar/gky1130 PMC6324026

[40] Sigrist, C. J. et al. PROSITE, a protein domain database for functional characterization and annotation. *Nucleic Acids Res.* **38**, D161–D166 (2010). 19858104 10.1093/nar/gkp885 PMC2808866

[41] Thomas, P. D. et al. PANTHER: a library of protein families and subfamilies indexed by function. *Genome Res.* **13**, 2129–2141 (2003). 12952881 10.1101/gr.772403 PMC403709

[42] Wang, K., Li, M. & Hakonarson, H. ANNOVAR: functional annotation of genetic variants from high-throughput sequencing data. *Nucleic Acids Res.* **38**, e164 (2010). 20601685 10.1093/nar/gkq603 PMC2938201

[43] Rentzsch, P., Schubach, M., Shendure, J. & Kircher, M. CADD-Splice-improving genome-wide variant effect prediction using deep learning-derived splice scores. *Genome. Med.* **13**, 31 (2021). 33618777 10.1186/s13073-021-00835-9 PMC7901104

[44] Gudmundsson, S. et al. Variant interpretation using population databases: Lessons from gnomAD. *Hum. Mutat.* **43**, 1012–1030 (2022). 34859531 10.1002/humu.24309 PMC9160216

[45] Lee, S. et al. Optimal unified approach for rare-variant association testing with application to small-sample case-control whole-exome sequencing studies. *Am. J. Hum. Genet.* **91**, 224–237 (2012). 22863193 10.1016/j.ajhg.2012.06.007 PMC3415556

[46] Lee, S., Teslovich, T. M., Boehnke, M. & Lin, X. General framework for meta-analysis of rare variants in sequencing association studies. *Am. J. Hum. Genet.* **93**, 42–53 (2013). 23768515 10.1016/j.ajhg.2013.05.010 PMC3710762

[47] Benjamini, Y. & Hochberg, Y. Controlling the false discovery rate: a practical and powerful approach to multiple testing. *J. R. Stat. Soc. Ser. B* **57**, 289–300 (1995).

